# Tip-enhanced Raman spectroscopic imaging of patterned thiol monolayers

**DOI:** 10.3762/bjnano.2.55

**Published:** 2011-08-30

**Authors:** Johannes Stadler, Thomas Schmid, Lothar Opilik, Phillip Kuhn, Petra S Dittrich, Renato Zenobi

**Affiliations:** 1 Department of Chemistry and Applied Biosciences, ETH Zurich, Wolfgang-Pauli-Strasse 10, HCI E 329, CH-8093 Zurich, Switzerland

**Keywords:** mercaptopyridine, microcontact printing, monolayer, spectroscopic imaging, tip-enhanced Raman spectroscopy

## Abstract

Full spectroscopic imaging by means of tip-enhanced Raman spectroscopy (TERS) was used to measure the distribution of two isomeric thiols (2-mercaptopyridine (2-PySH) and 4-mercaptopyridine (4-PySH)) in a self-assembled monolayer (SAM) on a gold surface. From a patterned sample created by microcontact printing, an image with full spectral information in every pixel was acquired. The spectroscopic data is in good agreement with the expected molecular distribution on the sample surface due to the microcontact printing process. Using specific marker bands at 1000 cm^−1^ for 2-PySH and 1100 cm^−1^ for 4-PySH, both isomers could be localized on the surface and semi-quantitative information was deduced from the band intensities. Even though nanometer size resolution information was not required, the large signal enhancement of TERS was employed here to detect a monolayer coverage of weakly scattering analytes that were not detectable with normal Raman spectroscopy, emphasizing the usefulness of TERS.

## Introduction

The chemical characterization of surface adsorbates is of great interest in several areas of research. The composition of biological membranes or of artificially structured surfaces, used in molecular electronics, determines their properties as well as their function. However, characterization is difficult due to the small size and the low number of the molecules that comprise these structures. Most techniques such as nuclear magnetic resonance (NMR), infrared (IR) spectroscopy and Raman spectroscopy (RS) lack the necessary spatial resolution, while others such as scanning tunneling microscopy (STM) or scanning electron microscopy (SEM) do not provide enough chemical information. Furthermore, the limited quantity of analyte results in weak signals rendering characterization even more difficult. Ideally, information should be gathered with minimal disturbance of the molecules, which rules out any kind of labeling, and also emphasizes the need for an ambient pressure technique.

Tip-enhanced Raman spectroscopy (TERS) has been developed to obtain chemical information with very high spatial resolution [1–4], or chemical information from very few molecules, and in some cases even single molecules [5–7]. The technique uses a metal or metalized AFM/STM tip to confine the laser energy focused by a confocal microscope objective and to act as a “nano-torch” to locally excite molecules underneath it and enhance their Raman signals. Here an application of TERS is demonstrated which particularly exploits the signal enhancing effect anywhere on the sample surface. Due to the increased signal, TERS can detect small amounts of analyte in a short time, allowing acquisition of Raman images of a surface area covered with weakly scattering molecules. The information from such a Raman image was used here to chemically identify and localize two different thiol isomers in an inhomogeneous self-assembled monolayer (SAM). In this work the lateral resolution of TERS was not used to its full potential, but, by exploiting the signal enhancement, weak scatterers could be identified over a larger area.

Thiols are used for several purposes. They can form a very thin protective layer on metal surfaces [8] or can be employed in sensorics [9–10]. Moreover, thiols have been suggested as components in molecular electronics [11]. Thiols are commercially available in a wide chemical diversity and can easily be linked to a variety of (bio-)molecules using simple chemistry. Thus, they may be used to pattern and functionalize entire surfaces or certain surface areas. Here, selected areas on a gold surface were modified by a thiol and, in a second step, the remaining substrate was covered by a secondary thiol film. This type of surface can act as a basis for biosensors [12–13].

To produce patterned SAM structures on a gold surface, microcontact printing is the tool of choice. The technique originated in the lab of Whitesides in 1993 [14] and provides cheap, quick and easy access to patterned surfaces after the initial production of a microfabricated master that can be moulded multiple times to create stamps. The pattern on the surface allows us in a first step to check whether or not a patterned region can be discerned from the bare substrate using TERS. In a further step, the functionalization of the bare substrate with a secondary analyte shows, that two very similar analytes can be differentiated and localized.

For our experiments we chose 2- and 4-mercaptopyridine (2-PySH and 4-PySH), which have been used to modify electrode surfaces in protein electrochemistry [15]. In a non-destructive experiment, the spectral signature of both isomers was employed to map their distribution on the sample surface using TERS in a gap mode configuration. The term “gap mode” signifies the use of a metal tip for TERS in close proximity (<5 nm distance) to a metal surface with the analyte in between the two. In this geometry, a very strong, highly localized electromagnetic field is formed in the small gap between the metal tip and the substrate, leading to a strong signal enhancement and a well-localized signal source [16–20]. The extent of the enhancement and, along with that, the intensity of the measured Raman signal strongly depends on the tip–surface distance [5,21–23]. Fluctuations in the tip–sample distance can lead to considerable Raman intensity changes, thus flat gold films are an ideal substrate to minimize the STM feedback changes and distance related artifacts.

Previous studies on self-assembled thiol films were conducted using AFM [13], STM [15,24–25], XPS and Ellipsometry [26] as well as Raman spectroscopy [27–28]. Single point TERS experiments have already been presented by our group in a study of the spectral and binding properties of 4-PySH on gold [29]. By using TERS, the topography and the chemical composition of molecular monolayers can be measured simultaneously during Raman imaging with high lateral resolution, around 15 nm, as demonstrated for areas of less than 500 × 500 nm^2^ [30]. As shown here, large sample areas can also be measured to gain information from coarser structures. Traditionally this is the scale where confocal Raman microscopy has its strengths, but due to the low signal intensity from molecular monolayers, an enhanced Raman technique is necessary to determine the chemical identity of the molecules. In surface-enhanced Raman spectroscopy (SERS) experiments (with a rough Ag film as a substrate, produced by vapor coating with randomly located enhancement hot-spots), the necessary enhancement can in principle be reached. An attempt was made to visualize the distribution of molecules using SERS, but this did not yield satisfactory results. However, the large signal-to-noise ratio in every pixel of a TERS image allows one to obtain enough information to distinguish a full monolayer from a few scattered molecules on the surface that contribute to the overall signal and thus allows the visualization of patterned monolayer structures.

In this article, we show that TERS can be used to image chemically heterogeneous surfaces without the need for labeling, even when the different adsorbates have very similar properties, i.e., a monolayer consisting of both 2-PySH and 4-PySH. With TERS, both isomers could be localized on the surface by their spectroscopic signature and approximate information on the surface coverage could be obtained from the intensity of characteristic marker bands within the spectra. Due to the strong enhancement, this could even be done with a (sub-)monolayer of non-resonant molecules. Similarly to AFM and STM, TERS images could be acquired with different step sizes to gather information about objects or structures on the nanometer or micrometer scale.

## Results and Discussion

**Figure 1:**
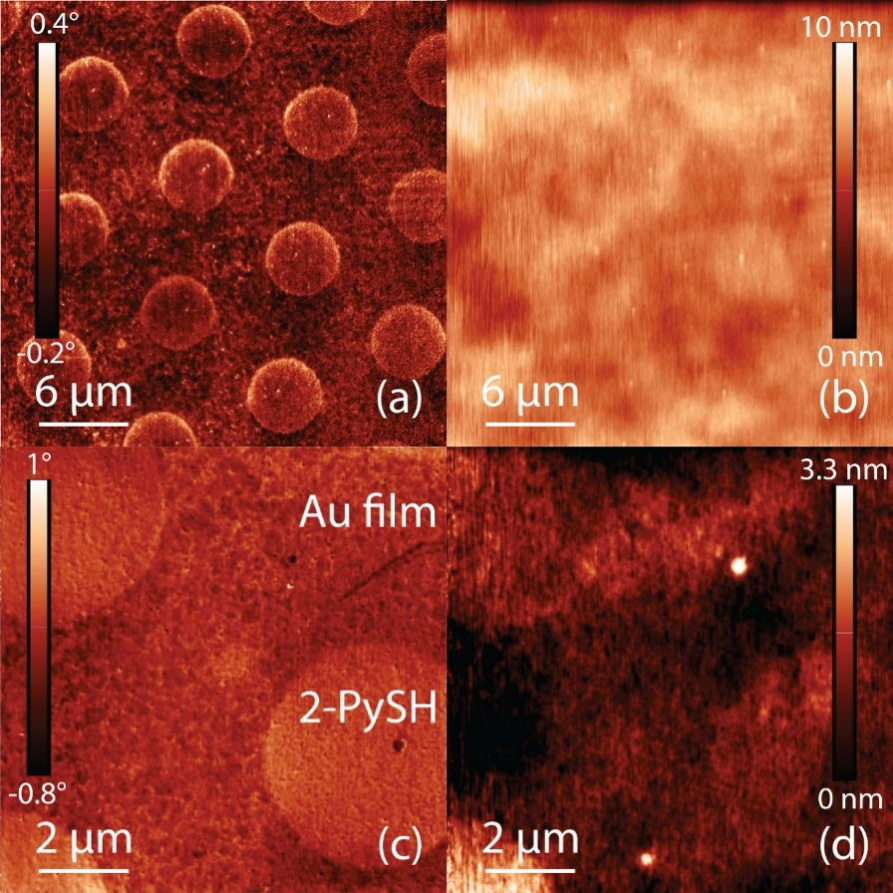
(a), (c) 30/10 μm AFM tapping mode phase images of microcontact printed 2-PySH on a gold surface. (b), (d) Corresponding height images showing slight elevation of the thiol layer, slightly masked by the system noise, suggesting a height between 0.5–1.0 nm. The phase image clearly illustrates the different surface properties of the printed thiol in comparison to the pure gold surface.

Flat template-stripped gold thin films with a thickness of 60–200 nm on glass were used as substrates for the experiments and functionalized with thiols using micro contact printing (for details see the Experimental section). In a first step, the efficiency and coherence of the transferred 2-PySH thiol monolayer was investigated using AFM. Figure 1 shows height and phase images of two AFM scans. The 30 μm overview scan in Figure 1a shows a clear phase difference between the bare gold surface and the circular microcontact printed thiol covered areas. The corresponding height image in (b) exhibits a similar pattern, but the small elevations from the thiol layer are somewhat masked by the system’s background noise. The enlarged 10 μm scan in (c) and (d) indicates a continuous smooth printing with few defects within the printed areas. The height signal now shows the elevation of the thiols a little more clearly, suggesting a step height <1 nm, in agreement with existing literature [31–33]. Due to the slight curvature of the underlying gold surface this value cannot be determined more exactly.

Attempts to spectroscopically visualize the thiol layer with confocal Raman spectroscopy failed due to the intrinsically weak cross section of the Raman process and the small number of molecules forming the investigated monolayer. No typical Raman bands were seen during excitation for 6 × 10 s at 3 mW by a 632.8 nm laser in confocal measurements. The absence of signals from decomposition products (carbonaceous decomposition products usually scatter strongly) leads to the conclusion that the SAM was not destroyed by the high laser power, but that the intensity of Raman signals from the intact monolayer was too weak to be detected.

In experiments on Ag SERS substrates (nominal thickness 6 nm), the printed patterns could not be detected and localized by confocal RS. Either no Raman signals at all or homogeneous signals from all over the substrate were detected (data not shown). Due to the strong but inhomogeneous enhancement by single sites on a typical SERS substrate, it is most likely that a very small fraction of molecules diffusing on the Ag surface during production of the samples dominated the spectra, preventing a localization of the molecules. Another possible explanation is that the roughness of the SERS substrate interfered with the patterning process used (compare Figure 6).

**Figure 2:**
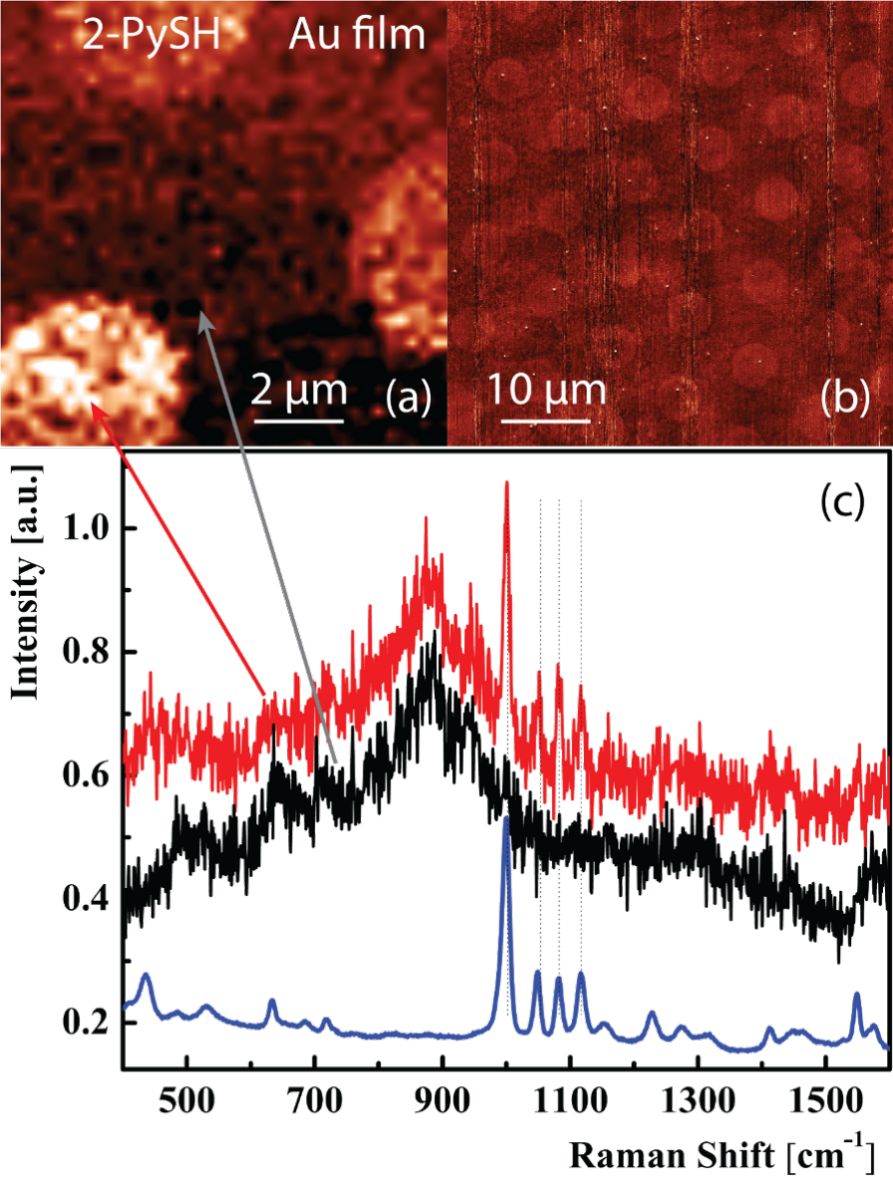
(a) 10 μm tip-enhanced spectroscopic image of microcontact printed 2-PySH on gold. Evaluation of a thiol marker band intensity at 1000 cm^−1^ yields a spot size of 5 μm with 4.1 μm spacings. (b) 50 μm phase image of the same area shows intact thiol structures after acquisition of the spectroscopic image in (a). (c) 120 s reference SERS spectrum of 2-PySH (blue) and 2 s TERS spectra from (a) on the thiol (red) and on the bare gold surface (black). Spectra have been offset (red) and rescaled (blue) for better visibility.

Results of a tip-enhanced Raman experiment are presented in Figure 2. An etched silver tip was used to probe the surface and enhance the Raman signals from the thiol monolayer. A 64 × 64 pixel map at 10 × 10 μm^2^, with 156 nm/pixel, and full spectral information at every pixel, was acquired with a 632.8 nm laser at a power of 300 μW and an acquisition time of 2 s per spectrum. Figure 2a shows the intensity distribution of the 2-PySH marker band at 1000 cm^−1^ indicating the presence of 2-PySH on the surface. The circular structures from the microcontact printing can be seen clearly and distinguished from the pure metal background. The experiment did not destroy the thiol on the surface due to the low laser power and the non-contact nature of the STM. The AFM phase image in Figure 2b, taken after the TERS map from the same sample region, still shows the intact thiol patterns. Figure 2c shows a 120 s reference SERS spectrum of 2-PySH (blue, rescaled) and two 2 s TERS spectra from the positions indicated in (a), taken on the thiol layer (red, offset for clarity) and on the bare gold surface (black). The spectra clearly show the typical signals of the 2-PySH in the red curve and their absence in the black curve. The spectral background is caused by the Ag TERS tip.

**Figure 3:**
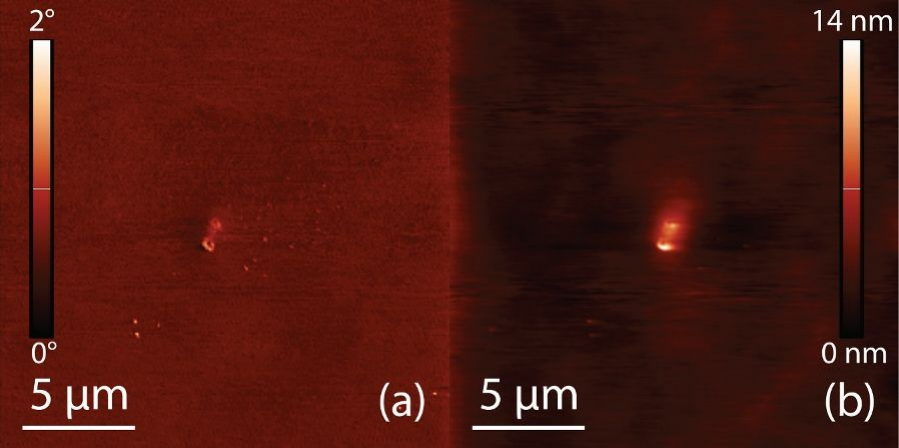
20 μm tapping mode AFM image of microcontact printed 2-PySH on gold immersed in 4-PySH to fill gaps. (a) Phase image; (b) Topography – no discernible patterns on the surfaces between the two thiols can be seen. The roughness in (a) shows the typical topographic structure of the underlying gold film. The central feature originates from an impurity on the sample surface. The height of the image (excl. the central feature) is around 2 nm.

A microcontact printed 2-PySH surface was then incubated in a 10 mM ethanolic solution of 4-PySH for 10 s, in order to fill the bare gold surfaces between the covered areas with 4-PySH. The result was a mixed monolayer of two thiols, with very similar properties, distributed on the surface in a well-defined pattern. The AFM (a) phase and (b) topography images in Figure 3 demonstrate that it is not possible to differentiate the two isomers on the surface based on these AFM experiments alone, due to their similar surface properties (height, friction).

**Figure 4:**
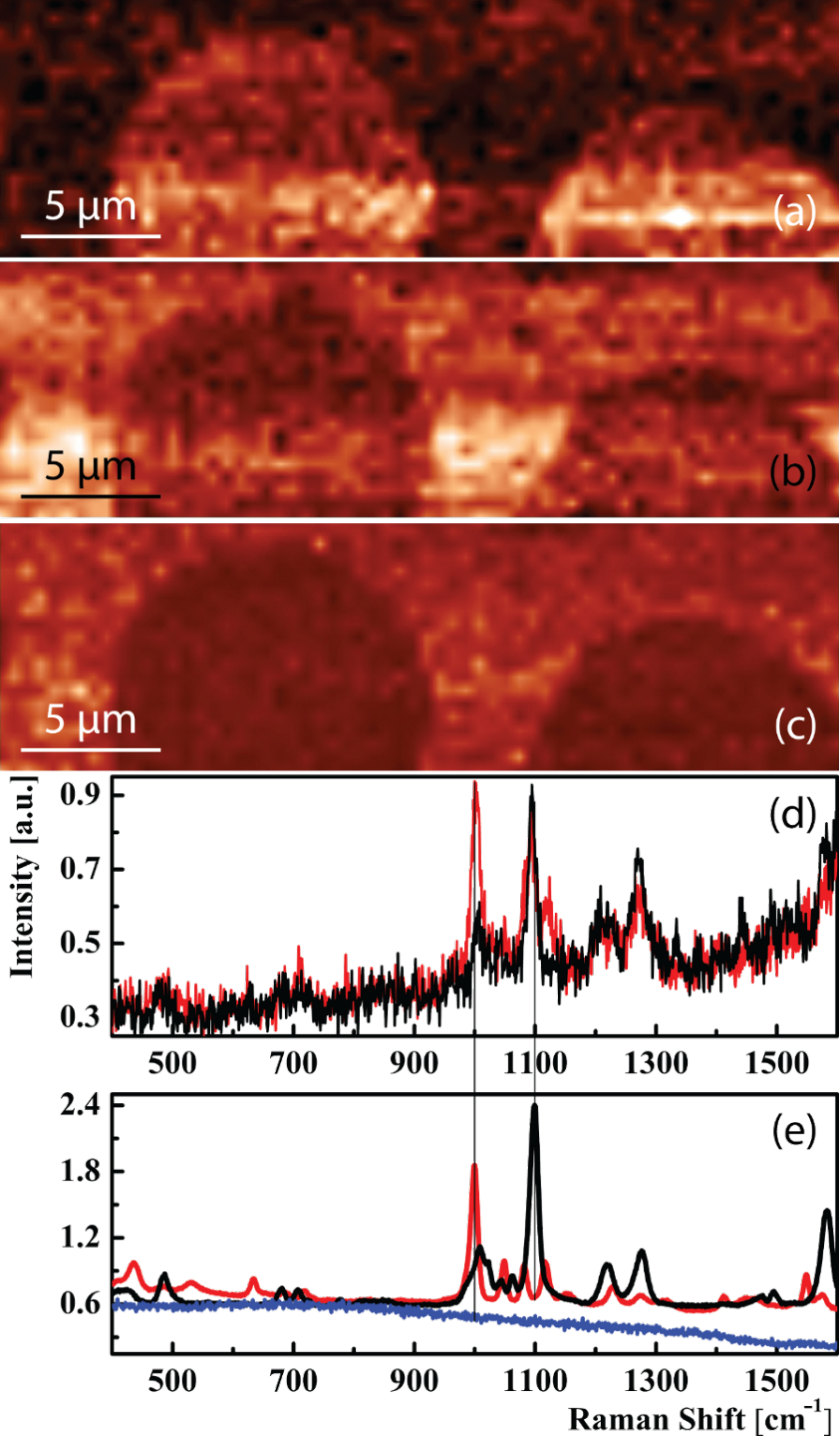
(a)–(c) 30 × 10 μm^2^ tip-enhanced spectroscopic images. (a) Intensity of the 2-PySH marker band at 1000 cm^−1^. (b) Intensity of the 4-PySH marker band at 1100 cm^−1^. Images show complementary patterns with higher intensity of the microcontact printed thiol in the circles and higher intensity of the thiol used to fill the gaps around the circular areas. (c) Intensity ratio of the two marker bands. (d) 0.5 s TERS spectra from the printed 2-PySH area (red) and the area covered with 4-PySH (black). Small residues of 4-PySH in the red curve are visible at 1100 cm^−1^. (e) 120 s reference SERS spectra from 2-PySH (red) and 10 s SERS spectrum of 4-PySH (black) and a 60 s confocal background spectrum (navy) from a 2-PySH thiol monolayer.

One possible but very tedious way to distinguish the two thiols would have been to use high resolution STM (which is usually size limited to the nanometer range) to search for typical molecular patterns in the SAM structure. However, both thiols can assemble in several different structures [15,24] and a possible mixing of both thiols would have led to further complications for STM. By using TERS imaging instead, and therefore gaining chemical contrast, the distribution of the two different thiols on the surface could be determined. Figure 4a and Figure 4b show the background corrected intensity maps of the 2-PySH and 4-PySH marker bands at 1000 cm^−1^ and 1100 cm^−1^, respectively. To exclude the possibility that changes in enhancement during the experiment are responsible for the contrast (e.g., due to changes in tip–sample distance), the ratio between the two marker bands is also shown in Figure 4c. A reasonably uniform distribution within the printed areas and only weak fluctuations of this ratio in the filled areas can be seen. The evaluation shows that the printed thiol patterns have a diameter of 12 μm and a 3.5 μm spacing, in agreement with the 12 μm hexagon with a 55% coverage of circles on the microcontact printing stamp (see Experimental). Figure 4d shows 0.5 s TERS spectra from the printed 2-PySH area (red) and the area filled with 4-PySH (black). All bands from the black spectrum correspond well with bands in the black 10 s reference SERS spectrum from 4-PySH. The red TERS spectrum from 2-PySH exhibits all the bands from the 120 s reference SERS spectra from 2-PySH, and also an additional peak at 1100 cm^−1^ from the strongest band of 4-PySH. Considering that during preparation the entire sample is immersed in 4-PySH to fill the gaps from the microcontact printing, a certain amount of 4-PySH is expected to be embedded in the area printed with 2-PySH. Additionally, the 60 s confocal background spectrum (navy blue) from the 2-PySH thiol monolayer shows that the Raman signal of the monolayer is too weak to be picked up by a confocal measurement.

## Conclusion

Full spectral imaging using TERS can be used to visualize the distribution of two very similar non-resonant thiols within a single monolayer on a gold film. Two isomeric thiol species were differentiated and localized on the sample surface using their spectroscopic signatures. The investigation of monolayers could be useful in the analysis of catalytic processes in heterogeneous catalysis [34], where the investigation of single active sites or the processes within a monolayer require signal enhancements from TERS combined with the ability to localize this enhancement on selected surface sites. The enhancement shown here can be conservatively estimated using the band intensity contrast between the confocal and the tip-enhanced case (<50 cts in 60 s confocally and 500 cts in 0.5 s for TERS), corrected by the area of origin known from previous experiments [30] (500 nm for confocal Raman and 25 nm in TERS), to be in the order of 10^5^–10^6^.

## Experimental

All spectra were acquired by a combined AFM/STM connected to a quadruple grating Raman spectrometer (NTMDT Ntegra Spectra, Zelenograd, Russia) coupled to an EMCCD (Andor Newton, Belfast, UK). This top-illumination TERS setup has been described in detail in [30].

Template-stripped gold films were created using a similar method to that described in [35], by coating polished Si(100) wafers (Si-Mat, Landsberg, Germany) in a Bal-Tec Med 020 coating chamber at pressures below 1 × 10^−5^ mbar, with gold (99.99%, Leica, Wetzlar, Germany) evaporated by resistive heating at a rate ≤ 0.1 nm/s. The deposited gold film was bonded to clean microscope slide fragments using NOA61 (Norland, Cranbury, USA). The gold films were mechanically stripped from the Si wafers and used immediately.

**Figure 5:**
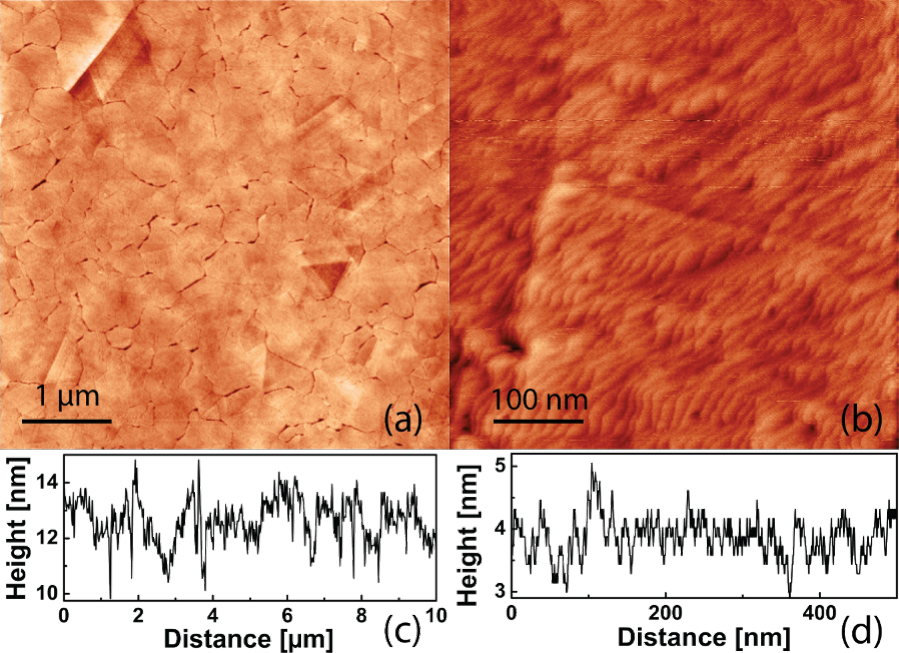
STM topography of a template-stripped gold surface (a) 10 × 10 μm^2^ topography scan showing single crystalline gold areas (top left) and a typical grain size of around 0.5 μm. (b) 500 × 500 nm^2^ image of the gold substrate showing slight corrugations. Line cuts (c) and (d) show peak-to-peak height differences of around 1–2 nm and a noise level below 1 nm.

STM was used to characterize the properties of the surface before sample preparation. The 10 × 10 μm^2^ STM scan in Figure 5a shows that during evaporation of the gold, under the given conditions, flat flakes of around 500 nm were formed, as well as some single crystalline domains (seen in the top left and right central area). A more detailed 500 nm scan in Figure 5b reveals small surface corrugations on a well ordered crystalline patch from Figure 5a with an average roughness of <1 nm in height.

**Figure 6:**
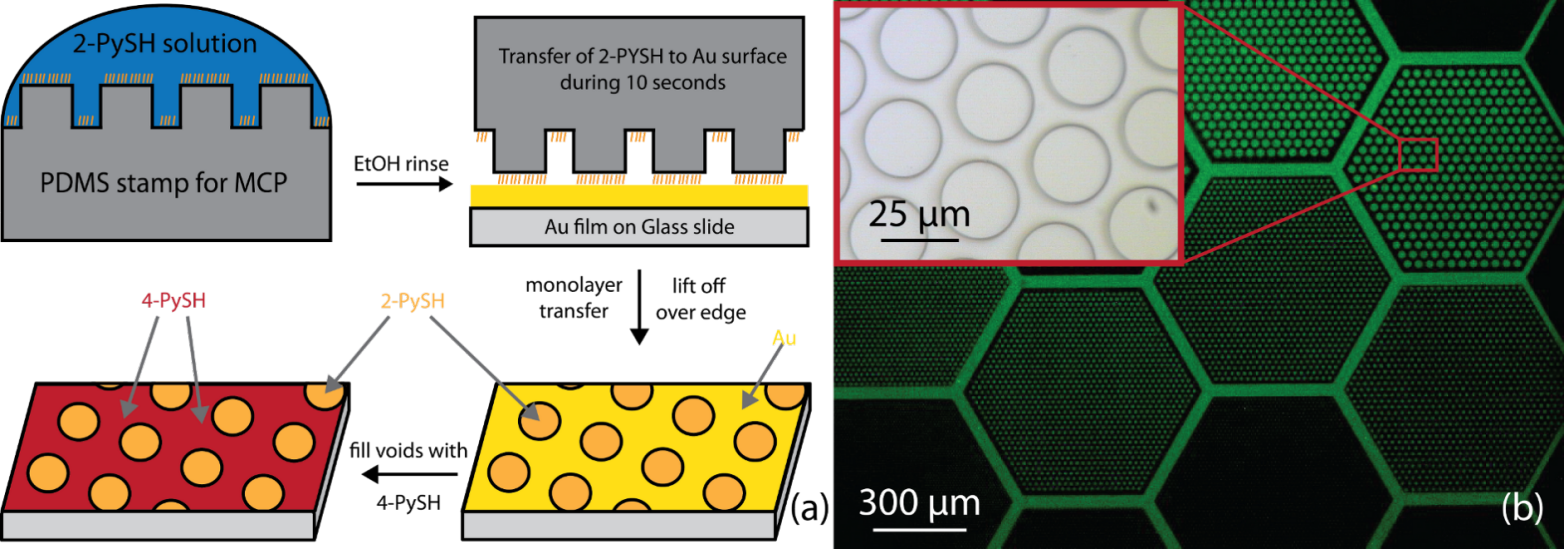
(a) Scheme of the microcontact printing process. Incubation of the stamp in ethanolic solution with subsequent printing on a template-stripped gold surface. (b) Magnified bright field image of a microcontact printing stamp for thiol deposition. The stamp consists of 650 μm hexagons filled with regular arrays of circles of different sizes and different distances between them. Circles from top to bottom have diameters of 25, 12 and 5 μm with increasing fill factors and distances from left to right. Inset: Magnified white light image from the circular elevations within a single hexagon.

Microcontact printing can be used to transfer a monolayer of thiols onto a noble metal substrate as described in [36–37]. A similar procedure to that described in [37] was used here to transfer a monolayer of 2-PySH onto the gold surface. To pattern the surface of the gold film, a microcontact printing stamp with differently sized elevated circles in hexagonal arrays was used (Figure 6). In Figure 6, a bright field image of the stamp layout is depicted. The stamp consists of an array of 650 μm wide hexagons, filled with flat circles of decreasing size (from top to bottom) and decreasing surface coverage (from left to right). The height of the elevated features of the stamp is defined by the thickness of the photoresist on the master (2.1 ± 0.1 μm).

The master for microcontact printing stamps was fabricated by standard photolithography [14,38–39]. Briefly, a positive resist (AZ1518) was spin-coated to a height of 2.1 ± 0.1 μm onto a silicon wafer, exposed through a sub-micrometer resolution chrome mask and developed. After overnight silanization, poly(dimethylsiloxane) (PDMS) was mixed in a 10:1 ratio with curing agent, poured onto the master, degassed and cured in an oven at 80 °C overnight. The cured PDMS mould was cut into stamps.

For microcontact printing, a droplet of a 10 mM ethanolic solution of 2-PySH was placed on the stamp for 1 min, and washed with copious amounts of ethanol. The pattern was then printed onto the gold film by placing the stamp with the pressure of its own weight onto the gold film for 10 s followed by careful lift-off.

The filling of the bare gold areas by 4-PySH was achieved by covering the entire printed gold film with a 10 mM ethanolic solution for 10 s followed by thorough cleaning with ethanol.

TERS tips were fabricated by electrochemical etching of silver tips (99.99% Ag wire, 0.25 mm, Aldrich) similarly to [20,40] in a solution of 1:1 to 1:2 (v:v) of perchloric acid (Riedel de Häen)/methanol with an etching voltage of 8 V. After etching, tips were rinsed with methanol to remove residues of the etchant. Etched tips were produced shortly before the experiments, and exposure to ambient conditions did not exceed 4 h. Tips with suitable shape for STM scanning as well as TERS activity were chosen by visual inspection under a 360× stereo microscope (Nikon, Amstelveen, Netherlands).

For the TERS experiments, tips were carefully approached to the sample and checked for Raman activity using a 632.8 nm Helium–Neon laser. Selected tips were then aligned using the laser scanning mirrors [30] and subsequently used for TERS mappings. For each TERS map, laser power and exposure time per spectrum were adapted according to the enhancement of the tip and the activity of the analyte. The laser power used ranged between 0.1–2.0 mW and collection times of 0.1–2.0 s per spectrum were chosen to yield a sufficient signal-to-noise ratio for the investigated Raman bands. Each pixel in the TERS experiments corresponds to one spectrum from an area of roughly 25 nm in diameter. In measurements with larger pixel to pixel distances, only the probed area contributed to the respective Raman spectrum.
